# Participation in activities of daily living after the Akwenda Intervention Program for children and young people with cerebral palsy in Uganda: A cluster‐randomized trial

**DOI:** 10.1111/dmcn.16258

**Published:** 2025-02-18

**Authors:** Elizabeth Asige, Gillian Saloojee, Godfrey Wanjala, Carin Andrews, Lukia H. Namaganda, Angelina Kakooza‐Mwesige, Diane L. Damiano, Hans Forssberg

**Affiliations:** ^1^ Department of Pediatrics and Child Health Makerere University Kampala Uganda; ^2^ CURIE Study Consortium Iganga‐Mayuge Health and Demographic Surveillance System Iganga Uganda; ^3^ Department of Physiotherapy, Faculty of Health Sciences University of the Witwatersrand Johannesburg South Africa; ^4^ Department of Women's and Children's Health Karolinska Institutet Stockholm Sweden; ^5^ Centre for Health and Sustainability, Department of Women's and Children's Health Uppsala University Uppsala Sweden; ^6^ Department of Epidemiology and Biostatistics Makerere University School of Public Health Kampala Uganda; ^7^ Rehabilitation Medicine Department, Clinical Center National Institutes of Health Bethesda MD USA; ^8^ Astrid Lindgren Children's Hospital Stockholm Sweden

## Abstract

**Aim:**

To evaluate the efficacy of the Akwenda Intervention Program on participation attendance and involvement of children and young people with cerebral palsy (CP) in rural Uganda.

**Method:**

This was a cluster‐randomized, controlled, single‐blind, interventional study of 100 participants with CP (aged 2–23 years; 48 females; allocated to the intervention or waiting list control group). Picture My Participation interviews assessed participation attendance and involvement in 20 home and community activities. Group differences were analysed using a Mann–Whitney *U* test and effect sizes were calculated. Change in attendance was related to age and functional level, and to improvements in child functioning, which was published in a previous report from the same study.

**Results:**

Attendance increased more in the intervention compared to the control group (*p* < 0.001; *r* = 0.48; *z* = −4.62) and across both Gross Motor Function Classification System (GMFCS) subgroups and two age subgroups (2–5 years and 13–23 years). Positive correlations were found between increases in attendance and higher GMFCS levels (*ρ* = 0.25, *p* = 0.03) and with all three caregiver assistance scales and the social function child scale of the Ugandan version of the Pediatric Evaluation of Disability Inventory. The intervention group had larger increases in involvement than the controls (*p* < 0.001; *r* = 0.41; *z* = −3.95), although positive changes were seen in both groups.

**Interpretation:**

The Akwenda Intervention Program, which intervened at the level of the child, family, and community, was successful in enhancing participation for children with CP.

## Graphical abstract

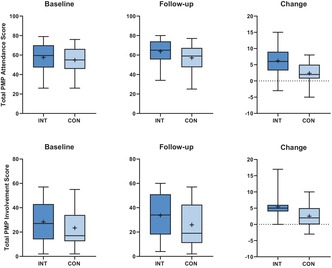

**Abbreviations**
PEDI‐UG: Ugandan version of the Pediatric Evaluation of Disability Inventory
PMP: Picture My Participation
TAD: technical assistive device

**What this paper adds**

- The Akwenda Intervention Program dramatically improved participation attendance and involvement in 20 home and community activities in children and young adults with cerebral palsy.
- Attendance increased for all functional levels but to a larger extent in those with greater mobility limitations.
- Participation increased over all activities but particularly in community activities.
- Improved social function and self‐care skills were associated with increased attendance frequency.

The perspective of childhood disability has shifted during the last decades from a biomedical to a broader, more holistic perspective that includes participation as an important factor, as well as acknowledging the influence of environmental and personal factors as expressed by the World Health Organization's International Classification of Functioning, Disability and Health.^1^ This ideological shift has transformed the care of children with disabilities to family‐centred and strength‐based practices aimed at improving participation and well‐being.^2^ However, a recent review of research into cerebral palsy (CP) in Africa found that most studies focused on body function and structures, suggesting that this shift in ideology and practice has not yet been disseminated.^3^ Living conditions for many children with disabilities in rural Africa are harsh because of stigma, neglect, and exclusion in addition to poverty and constrained resources.^4^, ^5^ We previously studied participation in children and young people with CP in rural Uganda and found lower attendance and involvement in activities of daily living compared to their peers without CP.^6^ Participation was particularly restricted for children with severe impairments and in community‐based activities. Other studies showed a higher prevalence of CP than in high‐income countries,^7^ poorer growth with more than two‐thirds being malnourished,^8^ poorer motor and social development,^6^, ^9^, ^10^ and a 25‐fold higher premature mortality than for those without CP.^11^ Access to habilitation services, including assistive devices, was poor; caregivers described financial hardship in seeking care for their child, inadequate knowledge on how to help their child, and lost hope for improvement.^9^

To meet the needs of these children and young people with CP and their families, we developed and implemented the Akwenda Intervention Program, a multi‐component intervention programme with three goals: (1) to improve child functioning, activity, and participation; (2) to increase caregivers' knowledge and skills, and improve their mental health and quality of life; and (3) to reduce stigma in the community and facilitate inclusion and participation. The Akwenda Intervention Program was developed by a team of international experts, and South African and Ugandan academic and health professionals.^12^ To improve participation, we wanted to include evidence‐based interventions promoting participation.

A literature search revealed few intervention studies in children with CP with a primary goal to increase participation in activities of daily living. A systematic review^13^ identified only seven randomized controlled trials with participation outcomes on a spectrum of childhood disabilities: none from low‐ to middle‐income countries and only two on CP, which included fitness and exercise interventions having only minimal positive effects on participation. Another systematic review^14^ identified eight intervention studies designed to increase participation in leisure time or habitual physical activity in children with CP, showing a small effect on habitual but not leisure physical activity. In an updated review^15^ on CP interventions, evidence was analysed for 15 goal areas, with none on participation.

Because we did not find any evidence‐based interventions specifically focused on improving participation in activities of daily living in children with CP, we sought guidance from conceptual papers on participation‐based therapy, which suggested that interventions should be goal‐oriented, family‐centred, collaborative, strength‐based, and undertaken in natural settings.^2^ These concepts were applied in the Akwenda Intervention Program; they are briefly described in the ‘Method’ section of the article and in more detail in Appendix S1 and previous publications.^12^, ^16^, ^17^

In this article, we report participation attendance and involvement assessed in 20 activities of daily living using the Picture My Participation (PMP) tool.^18^ The hypothesis was that participants in the intervention group would improve their attendance and involvement more than those in the control group. We also explored whether effects differed depending on age, sex, and functional limitations, and if there were associations between changes in participation and functional improvements reported previously.^16^

## METHOD

### Study setting and design

This is the second in a series of reports from a cluster‐randomized, controlled, assessor‐blinded study on a cohort of children and young people with CP, where one group received the intervention while the other served as a waiting list control.^12^, ^16^, ^17^ The study was conducted over 12 months (October 2021–September 2022) in the Iganga‐Mayuge Health and Demographic Surveillance Site, which covers a population of approximately 90 000 inhabitants in 65 villages in Eastern Uganda. Most residents were engaged in subsistence farming and living below or close to the poverty level. Detailed descriptions of the methods were published in a research protocol before study initiation,^12^ and in the first manuscript on gross motor function, mobility, self‐care skills, and social function outcomes.^16^

The study was approved by the Uganda National Council for Science and Technology (no. SS‐5173 & HS‐1979E) and School of Medicine and Ethics Committee (no. SOMREC 2021–273), and registered with the Pan‐African Clinical Trials Registry (PACTR202011738099314). All caregivers provided written informed consent; assent was obtained from the study participants when possible.

### Participants

A total of 100 children and young people (aged 2–23 years; 52 males) with a CP diagnosis confirmed by a child neurologist using the Surveillance of Cerebral Palsy in Europe definition were included in a CP cohort, previously identified and described in a series of studies.^19^ The cohort was divided in two arms: one group received the Akwenda Intervention Program and the other served as the control, waiting to receive the intervention the following year. Participants and their caregivers living in the same and neighbouring villages were clustered together in two groups stratified according to age, sex, and Gross Motor Function Classification System (GMFCS) level. A coin flip was used to decide which of the two groups should receive the intervention first. The demographic characteristics of both groups are presented in Table S1.

Ninety‐four participants completed the study: 48 in the intervention group and 46 in the control group (see the flow chart in Figure S1). In the intervention group, two participants withdrew, while three children in the control group were deceased and one child withdrew. The randomization process was successful in producing groups similar in all 11 baseline characteristics (*p* > 0.05; Table S1) and in gross motor function.^16^

### The Akwenda Intervention Program

For a detailed description of the Akwenda Intervention Program see Appendix S1 or the research protocol.^12^ The programme consisted of five intervention components; (1) caregiver‐led training workshops; (2) therapist‐led practical group sessions and workshops; (3) provision of technical assistive devices (TADs); (4) goal setting; and (5) communication and advocacy for behavioural and social change.^12^ The intervention was conducted in four caregiver groups over 12 months and included seven caregiver‐led workshops, 14 therapist‐led practical sessions, and four communication and advocacy sessions. Attendance was over 90% for all sessions. Caregivers attended sessions twice a month, and their children accompanied them to the second session each month. Transport reimbursement and meals were provided. In addition, 38 community stakeholders attended two sessions as part of the communication and advocacy for behavioural and social change. The intervention was coordinated by a physiotherapist/PhD student and implemented by a team of four caregiver facilitators, three part‐time therapists, a social worker, and a community mobilizer.

### Measurements

Outcome measures were collected at baseline and at follow‐up. The primary outcome measure was PMP, which measures attendance frequency and the level of involvement in 20 home and community activities.^18^ PMP was developed for use in low‐ and middle‐income countries, and was administered as an interview, with each item and response category illustrated using pictures. Attendance frequency was recorded using a 4‐point Likert scale for each of the 20 activities: 1 = never, 2 = seldom, 3 = sometimes, and 4 = always. Response alternatives were illustrated with baskets containing different amounts of fruit (empty to full); respondents were instructed to point to the appropriate basket. Respondents were subsequently asked about the child's involvement only in activities that scored as ‘always’ or ‘sometimes’ for attendance. Involvement was rated on a 3‐point Likert scale: 1 = not, 2 = somewhat, and 3 = very. These alternatives were also illustrated with pictures: (1) a child remaining by themselves; (2) looking at other children when playing; or (3) playing with other children. The internal consistency for the total scale was good (Cronbach's alpha = 0.85)^20^ and the test–retest reliability for the total scale showed moderate reliability (interclass correlation coefficient of 0.64 for attendance and 0.63 for involvement).^21^ We used the caregiver's version of the PMP for consistency of reporting because approximately half of the participants with CP were unable to comprehend and respond accurately because of cognitive or communication impairments.^18^, ^22^ The caregiver version of the PMP was pre‐tested, adjusted, and used in a previous study describing participation in a population‐based cohort of children and young people with CP and their peers without CP before its use in this study.^6^

Interviews (lasting 30–40 minutes) were conducted in the home environment in the local language (Lusoga) by social workers living in the area and familiar with the culture and language. The assessor performing the follow‐up interview was blinded to group allocation. Both interviewers received study‐specific training to ensure data consistency and quality. Responses were recorded electronically using a computer tablet and the Open Data Tool Kit (https://codeforaotearoa.github.io/). Data entry was reviewed for completeness and quality while the assessor was still in the field.

Sociodemographic information and clinical data, including anthropometry and GMFCS levels,^23^ were collected before group allocation. GMFCS classifies mobility on a 5‐point ordinal scale, from level I (independent) to level V (totally dependent on assistance). The 66‐item Gross Motor Function Measure^24^ and the Ugandan version of the Pediatric Evaluation of Disability Inventory (PEDI‐UG)^25^ were used to measure changes in the child's gross motor function, mobility, self‐care, and social function, and the need for caregiver assistance, with outcomes reported previously.^16^ They are used in the current article to explore the relationships between intervention effects on functioning and participation.

### Statistical analysis

Data were analysed using SPSS v29.01.0 (IBM Corp., Armonk, NY, USA). Pearson *χ* ^2^ tests were performed at the statistical significance level of *p* < 0.05 to assess group differences across categorical variables describing participant baseline characteristics.

The total attendance score was calculated for each participant by adding up all attendance scores (never = 1, seldom = 2, sometimes = 3, always = 4) for the 20 activity items, with a range from 20 to 80. We report the median and interquartile range (IQR) of the total attendance score at baseline and follow‐up, and of the change score from baseline to follow‐up. To assess whether effects between groups differed according to age or functional level, participants were subdivided into three age subgroups (2–5 years, 6–12 years, and 13–23 years) and two GMFCS subgroups (GMFCS levels I and II, GMFCS levels III–V). An independent‐samples Mann–Whitney *U* test was used to test differences between groups; effect sizes were estimated using the formula *r* = *z*/sqrt (*n*) (*z* is the standardized test statistics [SPSS] and *n* is the number of observations).^26^ A Wilcoxon signed‐rank test was used to explore differences across time points in the groups.

Spearman's rank correlation coefficient analysis was used to correlate the total attendance change score with age and GMFCS level. To evaluate whether the amount of change in attendance was related to sex, we compared change scores across sexes using a Mann–Whitney *U* test. Pearson's rank correlation was used to relate the total attendance change score of each child to their change scores for the 66‐item Gross Motor Function Measure^24^ and PEDI‐UG scores,^25^ which were reported previously.^16^

To explore whether attendance was differentially influenced across the 20 activity items, we calculated the activity attendance score for each activity item by adding the attendance scores of all participants per group at baseline and at follow‐up, respectively. The activity attendance change score was calculated as the difference between the two assessments, normalized by dividing it by the number of participants assessed.

Involvement scores were only collected for activities scored as ‘always’ or ‘sometimes’ for attendance. The total involvement score was calculated for each participant as the sum of the scores (not = 1, somewhat = 2, very = 3). A Mann–Whitney *U* test was used to test differences between groups and *r* = *z*/sqrt (*n*) to estimate the effect size.

To explore whether the intervention affected involvement differentially across activity items, we calculated the activity involvement score for each activity item by adding the involvement scores of all participants. We only included participants who attended the particular activity always or sometimes at both assessments. The activity involvement change score was calculated as the difference between the two assessments, normalized by dividing it by the number of participants assessed for that item.

## RESULTS

### Attendance

**FIGURE 1:**
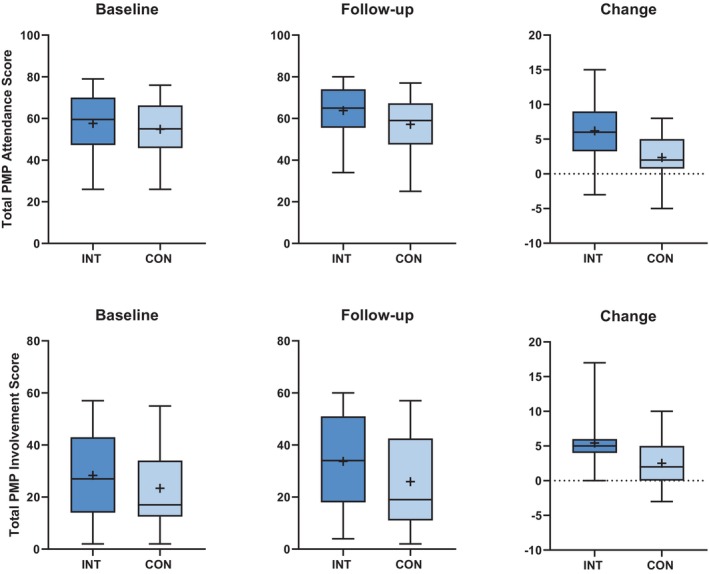
Median, IQR, and range of total attendance and total involvement scores at baseline and follow‐up and for the change score (difference between baseline and follow‐up) of the intervention (dark shade) and control (light shade) groups. An independent‐samples Mann–Whitney *U* test revealed no difference between groups at baseline (attendance *p* = 0.298; involvement *p* = 0.145) but significant differences between groups at follow‐up (attendance *p* = 0.013; involvement *p* = 0.020) and for the change scores (both scores *p* < 0.001). Abbreviation: PMP, Picture My Participation.

The total attendance score of the intervention and control groups, and their respective age and GMFCS subgroups, are shown in Table 1 and Figure 1. The change score was greater in the intervention group (median change = 6.0; IQR = 3.25–9.0) than in the control group (median change = 2.0; IQR = 0.75–5.0; *p* = 0.001; *r* = 0.48; *z* = −4.624). There were also group differences between GMFCS subgroups and the 2 to 5 years and 13 to 23 years subgroups, but not the 6 to 12 years subgroup. There was a weak positive correlation between total attendance change score and GMFCS level (correlation coefficient = 0.251, *p* = 0.025; Table 2), but none with age. Change scores did not differ across sexes (*p* = 0.17). Thus, the intervention had an impact on both younger and older participants and on all GMFCS levels, with a larger effect on those with the greatest mobility limitations.

**TABLE 1:** Total attendance score (median and interquartile range) across all 20 activities (range: 20–80) for each participant at baseline and at follow‐up for participants of the intervention and control groups and for their age and GMFCS subgroups.

Total attendance score	Intervention group, *n* = 48					Control group, *n* = 46					
	*n*	Baseline	Follow‐up	Change	*p*	*n*	Baseline	Follow‐up	Change	*p*	*p*
All	48	59.5 (47.25–70.0)	65.0 (55.5–74.0)	6.0 (3.25–9.0)	< 0.001	46	55.0 (45.75–66.25)	59.0 (47.5–67.25)	2.0 (0.75–5.0)	< 0.001	< 0.001
2–5 years	10	56.5 (47.75–59.75)	61.0 (57.25–65.0)	7.5 (3.75–9.5)	0.005	7	55.0 (49.0–60.0)	58.0 (53.0–60.0)	4.0 (0.0–5.0)	0.058	0.050
6–12 years	23	63.0 (45.0–74.0)	68.0 (50.0–76.0)	5.0 (1.0–11.0)	< 0.001	25	52.0 (45.5–65.0)	57.0 (48.5–66.0)	3.0 (1.0–5.0)	< 0.001	0.157
13–23 years	15	61.0 (43.0–70.0)	67.0 (47.0–76.0)	6.0 (4.0–9.0)	< 0.001	14	58.5 (42.0–71.0)	62.0 (42.75–71.0)	1.5 (−0.25 to 3.0)	0.048	0.003
GMFCS levels I and II	26	68.5 (61.0–76.25)	73.5 (67.0–77.25)	5.0 (1.75–7.5)	< 0.001	19	66.0 (62.0–75.0)	67.0 (62.0–75.0)	1.0 (0–3.0)	0.008	0.003
GMFCS levels III, IV, and V	22	46.0 (35.5–56.5)	54.0 (46.5–62.25)	7.0 (4.0–11.0)	< 0.001	27	50.0 (41.0–55.0)	53.0 (41.0–55.0)	3.0 (1.0–5.0)	< 0.001	< 0.001

*Note*: The change score was the difference between the baseline and follow‐up scores. A Wilcoxon signed‐rank test was used to study differences over time in the groups (the *p*‐value at the end each group). A Mann–Whitney *U* test was used to test differences in change scores between groups (the *p*‐values in the far right column; significance level set at *p* = 0.05).

Abbreviation: GMFCS, Gross Motor Function Classification System.

**TABLE 2:** Correlation coefficient and associated *p*‐values between the total attendance change score, and the change scores for the GMFM‐66, and PEDI‐UG mobility, self‐care, and social function on both child and caregiver assistance scales, and with age and GMFCS subgroups for the entire population (both groups).

	Correlation coefficient	*p*
GMFM‐66	0.136	0.191
Mobility	−0.008	0.935
Self‐care skills	0.109	0.298
Social function	0.265	0.010
Caregiver assistance mobility	0.245	0.018
Caregiver assistance self‐care skills	0.309	0.003
Caregiver assistance social function	0.325	0.001
Age	−0.023	0.830
GMFCS	0.251	0.015

*Note*: The Pearsons's rank correlation coefficient was used to analyse the GMFM‐66 and PEDI‐UG scores. The Spearman's rank correlation coefficient was used to analyse age and GMFCS.

Abbreviations: GMFCS, Gross Motor Function Classification System; GMFM‐66, 66‐item Gross Motor Function Measure; PEDI‐UG, Ugandan version of the Pediatric Evaluation of Disability Inventory.

**FIGURE 2:**
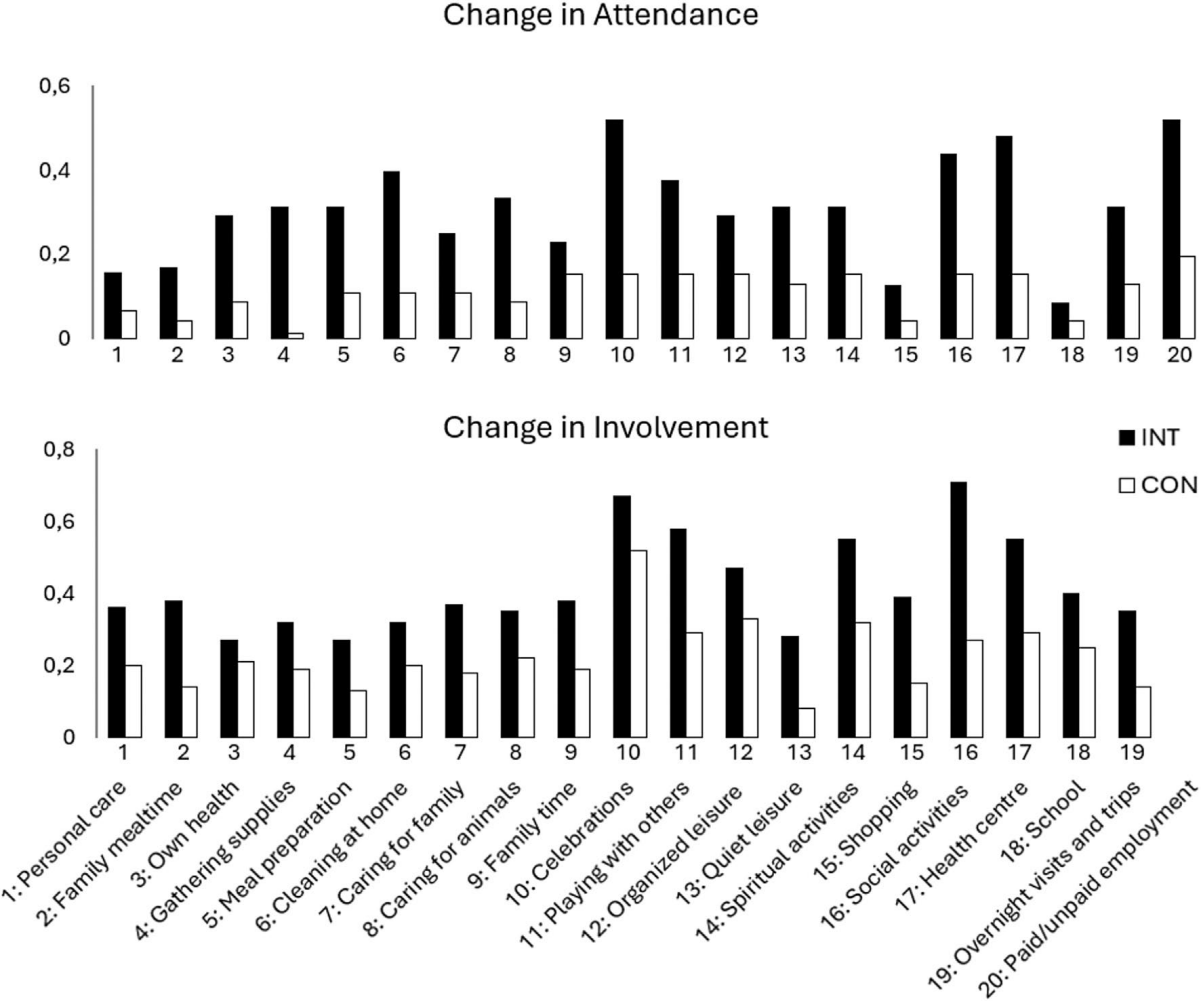
The activity attendance change scores and the activity involvement scores of the intervention (INT; black) and control (CON; white) groups are shown across the 20 activities for attendance and 19 activities for involvement excluding ‘paid/unpaid employment’ due to few particpants attending always or sometimes. The histograms show the normalized change score for each activity, that is, the difference between the activity attendance score and activity involvement score respectively, at baseline and at follow‐up, divided by the number of participants assessed.

The change in attendance is explored in Table 3 and Figure 2, showing increased attendance in all activities but to varying degrees across activities and between groups. The activity attendance change scores increased in all activities in the intervention group with normalized change scores ranging from 0.08 (school) to 0.52 (celebrations, employment) points. The activity attendance change scores for the control group also increased in most activities, but to a smaller extent, ranging from 0.04 (school, shopping, family mealtime) to 0.20 (employment) points.

**TABLE 3:** Attendance in each of the 20 activities of the intervention and control groups.

Activity item in PMP	Intervention group, *n* = 48					Control group, *n* = 46				
	Baseline always/sometimes	Follow‐up always/sometimes	Baseline activity attendance score	Follow‐up activity attendance score	Activity attendance change score	Baseline always/sometimes	Follow‐up always/sometimes	Baseline activity attendance score	Follow‐up activity attendance score	Activity attendance change score
1: Personal care	47	48	185	191	6	44	45	172	175	3
2: Family mealtime	45	48	182	190	8	45	44	175	177	2
3: Own health	41	47	170	184	14	33	36	143	147	4
4: Gathering supplies	31	33	130	145	15	23	21	108	114	6
5: Meal preparation	41	43	164	179	15	39	40	160	165	5
6: Cleaning at home	38	42	154	173	19	35	38	142	147	5
7: Caring for family	41	44	169	181	12	40	39	157	162	5
8: Caring for animals	30	33	133	149	16	24	25	110	114	4
9: Family time	45	47	176	187	11	42	43	164	171	7
10: Celebrations	25	31	120	145	25	24	24	112	119	7
11: Playing with others	40	41	155	173	18	34	36	149	156	7
12: Organized leisure	20	24	103	117	14	12	17	92	99	7
13: Quiet leisure	36	38	149	164	15	37	38	147	153	6
14: Spiritual activities	30	29	125	140	15	26	25	119	126	7
15: Shopping	29	30	129	135	6	27	28	124	126	2
16: Social activities	15	22	95	116	21	12	14	83	90	7
17: Health centre	38	42	148	171	23	28	34	134	141	7
18: School	15	16	93	97	4	8	8	69	71	2
19: Overnight visits, trips	26	27	120	135	15	21	24	105	111	6
20: Paid/unpaid employment	5	9	64	89	25	3	4	55	64	9
Total of all 20 activities/maximum score	638/960	695/960	2764/3840	3061/3840	297	561/920	586/920	2520/3680	2628/3680	108

*Note*: Columns 2 and 3 of the table show the number of participants who scored always or sometimes at baseline and at follow‐up respectively; the next two columns present the attendance activity scores, which are the sum of the attendance (never = 1, seldom = 2, sometimes = 3, always = 4) of all participants in the group for each activity item. The change score is the difference in attendance activity score between baseline and follow‐up. The last row shows the sum of all 20 activities divided by the maximum value.

Abbreviation: PMP, Picture My Participation.

### Correlations between changes in attendance and child functioning

There were weak positive correlations between the total attendance change score and all three PEDI‐UG caregiver assistance scales and PEDI‐UG social function scale change scores (Table 3). There were no or negligible correlations between changes in attendance frequency and changes in mobility, self‐care skills, and the 66‐item Gross Motor Function Measure.

### Involvement

The total involvement change score was larger in the intervention group (median = 5.0; IQR = 4.0–6.0) than in the control group (median = 2.0; IQR = 0–5.0) (Figure 1; *p* = 0.001; *r* = 0.41; *z* = −3.947).

The change in involvement across 19 activities is shown in Table 4 and Figure 2. Increases in the normalized activity involvement change scores were observed in both groups but were greater in the intervention group (range from 0.28 [quiet leisure] to 0.71 [social activities]) than in the control group from 0.13 (meal preparation) to 0.52 (celebrations).

**TABLE 4:** Involvement at baseline and at follow‐up in the intervention and control groups.

Activity item in the PMP	Intervention group					Control group				
	Attendance at both assessments A/S, *n*	Baseline activity involvement score	Follow‐up activity involvement score	Change activity involvement score	Normalized change score	Attendance at both assessments A/S, *n*	Baseline activity involvement score	Follow‐up activity involvement score	Change activity involvement score	Normalized change score
1: Personal care	47	101	118	17	0.36	44	82	91	9	0.20
2: Family mealtime	45	107	124	17	0.38	43	91	97	6	0.14
3: Own health	41	94	105	11	0.27	33	68	75	7	0.21
4: Gathering supplies	31	70	80	10	0.32	21	50	54	4	0.19
5: Meal preparation	41	83	94	11	0.27	38	70	75	5	0.13
6: Cleaning at home	38	76	88	12	0.32	35	63	70	7	0.20
7: Caring for family	41	70	85	15	0.37	39	67	74	7	0.18
8: Caring for animals	29	59	69	10	0.35	23	45	50	5	0.22
9: Family time	45	110	127	17	0.38	42	90	98	8	0.19
10: Celebrations	24	45	61	16	0.67	23	36	48	12	0.52
11: Playing with others	40	82	105	23	0.58	34	78	88	10	0.29
12: Organized leisure	19	40	49	9	0.47	9	16	19	3	0.33
13: Quiet leisure	36	86	96	10	0.28	36	73	76	3	0.08
14: Spiritual activities	29	58	74	16	0.55	22	43	50	7	0.32
15: Shopping	28	60	71	11	0.39	27	48	52	4	0.15
16: Social activities	14	29	39	10	0.71	11	21	24	3	0.27
17: Health centre	38	72	93	21	0.55	28	50	58	8	0.29
18: School	15	38	44	6	0.40	8	21	23	2	0.25
19: Overnight visits and trips	26	45	54	9	0.35	21	37	40	3	0.14
20: Paid/unpaid employment^a^	5	5	9	4		3	4	4	0	
Pooled scores across activities	638/960	1330	1585	255	0.46	561/920	1053	1166	113	0.19

*Note*: Involvement at baseline and at follow‐up in the intervention and control groups are shown as the activity involvement score for each activity in which the involvement scores (not = 1, somewhat = 2, very = 3) are added for each participant attending that activity always or sometimes (A/S) at both baseline and follow‐up assessments. The first column for each group shows the number of participants attending always or sometimes at both assessments. The activity involvement change score is the difference between the two assessments. The normalized activity change score was calculated by dividing the change score by the number of participants present at both assessments.

Abbreviation: PMP, Picture My Participation.

^a^ Normalized change score was not calculated due to the low numbers attending both assessments.

## DISCUSSION

The results clearly show that the Akwenda Intervention Program improved participation across 20 activities of daily living for children and young people with CP living in a resource‐constrained environment in rural Uganda. Participants receiving the intervention increased their frequency of attendance and level of involvement more than those in the waiting list control group. Frequency of attendance increased regardless of GMFCS level but with larger effects for participants with greater mobility limitations. Positive effects were seen across all activity items but were stronger in community activities. Improvements in participation attendance were associated with improvements in child functioning, demonstrating the bidirectional nature of the different domains of the International Classification of Functioning, Disability and Health.

In Uganda, as in many African countries, a major factor limiting participation is negative social attitudes and stigma, viewing children with disabilities as bringing shame to the family and that they should be hidden from the public. These attitudes are driven by multiple personal and societal factors, ranging from misconceptions concerning the cause of the disability (e.g. a curse or punishment from the gods), the competence of individuals with disabilities, or discriminatory practices towards those with disabilities,^5^, ^27^ resulting in isolation and marginalization of children with disabilities and their families. Improving participation for these children and young people requires reducing stigma and improving inclusion. In the Akwenda Intervention Program, we applied several strategies for this. The communication and advocacy for social and behavioural change consisted of caregiver sessions, radio talk shows, and two sessions with key community stakeholders.

Caregivers were educated regarding the rights of children, possible causes of the disability, how to support and promote their child's development and well‐being, and how to identify barriers to participation and form action plans to overcome these barriers. Equipped with this new knowledge, caregivers' ability to discern specific needs and provide appropriate support to their child may have enabled them to transition from hopelessness to heightened expectations about the child's future.^28^ Interactions and sharing with other caregivers with similar experiences let them know they were not alone, helping them alleviate the stigma and shame of having a child with CP.^29^ In addition, the two communication and advocacy sessions for key individuals in the community (leaders, administrators, teachers, health staff) informed them about disability, stigma, human rights, and the barriers children and their families were facing. In a joint session, caregivers presented their plan on how to facilitate inclusion of these key individuals. The new knowledge of both caregivers and key community members probably changed attitudes and improved participation.

Other key components were goal setting, in which therapists and caregivers collaboratively identified goals for enhancing participation in activities of daily living, and provision of TADs for positioning and mobility. Provision of wheelchairs, in combination with activity and participation goals, allowed children with severe functional limitations to achieve a sitting position and move around, thus making it possible for them to get to new places and participate in activities of daily living instead of spending their time in one place, often lying down on a mat unable to change position or environment.^30^

In our previous PMP study, the largest differences between children with and without CP were in community activities.^6^ In this study, we used descriptive analyses to explore whether the intervention had an impact on certain activity items more than others. Attendance increased across all activity items but to varying degrees. Notably, of the top six activities exhibiting the largest change score, five were community activities (celebrations, employment, social activities, health centre, playing with others). A similar profile was seen in improved involvement.

Participation is influenced by the body structure, function, and activity limitations of the child.^31^, ^32^ We previously reported large improvements in social function and self‐care skills, and in all caregiver assistance scales of the PEDI‐UG.^16^ In this study, we found a direct correlation between these improvements and improvements in attendance frequency. This correlation does not indicate causality, meaning that either improvements in child functioning can promote greater participation or vice versa, or both.^33^

The challenges and needs across a population of children and young people with CP are broad and dependent on functional limitations and age. Yet, our results suggest that the Akwenda Intervention Program improved participation attendance across age and functional levels, although the results from the correlation indicated that the impact on attendance was greater in children with greater mobility limitations. One explanation for this could be the low baseline levels of participation in this subgroup^6^ because of widespread stigma in the communities and no access to TADs.

The difference in the change score of total attendance was large, *r* = 0.48 (equivalent to Cohen's *d* = 1.09), indicating that the intervention had a big impact. Compared to other studies reviewed,^13^, ^14^, ^34^ the Akwenda Intervention Program had a greater impact. One reason is probably the extremely poor situation of these children and families resulting in severely restricted attendance in all 20 activities.^6^ The children and families in our study were intervention‐naive with very few or none receiving an intervention before the study, while children with disability living in high‐income countries probably received a range of interventions and support before the intervention.

Evaluation of real‐world, complex, multi‐component interventions is methodologically challenging in ways not fully addressed in guidelines for non‐complex clinical trials.^17^ We used a randomized controlled trial design to alleviate confounding and selection bias and randomized participants into two equivalent geographical clusters regarding a range of baseline characteristics (Table S1). We selected outcome measures carefully to evaluate the goals of the intervention and used the PMP tool because it has been developed for low‐ and middle‐income countries and measures both attendance frequency and level of involvement. A limitation of the PMP is the moderate test–retest reliability.^21^ We did not test for interrater reliability; however, new testers were instructed and checked by the first assessor. We used blinded assessors; however, because of geographical clustering and other clues (e.g. TADs), blinding might have been compromised. Attrition was small in both groups and caregiver attendance at the intervention group sessions was high (> 90%). By following these rigorous procedures, we argue that the large and significant differences between groups can be attributed to the intervention, and that it markedly improved the participation of the children and young people with CP.

### Conclusions

The Akwenda Intervention Program promoted large increases in attendance frequency and the level of involvement in 20 activities of daily living, particularly in community activities and in children with greater mobility limitations. The effect was achieved by a combination of components, including educating and supporting caregivers, providing TADs, improving child functioning, and informing key individuals in the community. Hopefully, the significant impact of this participation‐based programme will inspire the development of similar efforts to improve participation and well‐being in other low‐ and middle‐income countries.

## Supporting information


**Table S1:** Baseline characteristics of children and young people with cerebral palsy and their primary caregivers.

**Figure S1:** Study flow chart.

**Appendix S1:** Implementation of the Akwenda Intervention Program for children and young people with cerebral palsy.

## Data Availability

The data that underlie the results reported in this article are described at the Swedish National Data Service. Data are made available upon request after ensuring compliance with relevant legislation. Data repository: https://doi.org/10.48723/enwx‐zn78
