# Supplementary material for: Participation in activities of daily living after the Akwenda Intervention Program for children and young people with cerebral palsy in Uganda: A cluster‐randomized trial
Source: Dev Med Child Neurol. 2025 Feb 18;67(9):1195–205. doi: 10.1111/dmcn.16258 (PMC12336405; doi:10.1111/dmcn.16258)
Supplement: Supplementary file 1 — Table S1: Baseline characteristics of children and young people with cerebral palsy and their primary caregivers. [file DMCN-67-1195-s001.docx]

Supplement Table 1

| **Baseline characteristics of children and young people**  **with cerebral palsy and their primary caregivers** | | | | | | |
| --- | --- | --- | --- | --- | --- | --- |
| **Category** | | **All**  **N=94 (100%)** | **Intervention**  **N=48 (100%)** | **Control**  **N=46 (100%)** | **Pearson chi (X^2^)** | **P-value** |
| Age | 2-5 years | 17 (18%) | 10 (21%) | 7 (15%) | 0.605 | 0.739 |
|  | 6-12 years | 48 (51%) | 23 (48%) | 25 (54%) |  |  |
|  | 13-23 years | 29 (31%) | 15 (31%) | 14 (30%) |  |  |
| Sex | Female | 42 (45%) | 19 (40%) | 23 (50%) | 1.031 | 0.310 |
|  | Male | 52 (55%) | 29 (60%) | 23 (50%) |  |  |
| Residence area | Semi-urban | 27(29%) | 13 (27%) | 14 (30) | 0.129 | 0.720 |
|  | Rural | 67 (71%) | 35 (73%) | 32 (70%) |  |  |
| Primary caregiver | Mother | 50 (53%) | 28 (58%) | 22 (48%) | 4.432 | 0.618 |
|  | Grandmother | 25 (27%) | 10 (21%) | 15 (33%) |  |  |
|  | Father | 13 (14%) | 7 (15%) | 6 (13%) |  |  |
|  | Other | 6 (6%) | 3 (6%) | 3 (6%) |  |  |
| Primary caregiver occupation | Subsistence farmer | 48 (51%) | 24 (50%) | 24 (52%) | 5.375 | 0.146 |
|  | Petty trade | 33 (35%) | 18 (38%) | 15 (33%) |  |  |
|  | Formal employed | 4 (4%) | 3 (6%) | 1 (2%) |  |  |
|  | Others | 9 (10%) | 3 (6%) | 6 (13%) |  |  |
| GMFCS level  2021 | I | 25 (27%) | 16 (33%) | 9 (20%) | 6.608 | 0.158 |
|  | II | 20 (21%) | 10 (21%) | 10 (22%) |  |  |
|  | III | 9 (10%) | 3 (6%) | 6 (13%) |  |  |
|  | IV | 21 (22%) | 8 (17%) | 13 (28%) |  |  |
|  | V | 19 (20%) | 11 (23%) | 8 (17%) |  |  |
| Marital Status | Married | 64 (68%) | 33 (69%) | 31 (67%) | 0.802 | 0.670 |
|  | Separated | 20 (21%) | 11 (23%) | 9 (20%) |  |  |
|  | Widowed | 10 (11%) | 4 (8%) | 6 (13%) |  |  |
| Level of Education | Unknown | 10 (11%) | 7 (14%) | 3 (7%) | 9.424 | 0.224 |
|  | None | 5 (5%) | 1 (2%) | 4 (9%) |  |  |
|  | Primary | 53 (56%) | 24 (50%) | 29 (63%) |  |  |
|  | Secondary | 18 (19%) | 10 (21%) | 8 (17%) |  |  |
|  | Tertiary/University | 8 (9%) | 6 (13%) | 2 (4%) |  |  |
| Monthly income Ugandan shs | < 100000 | 55 (59%) | 29 (60%) | 26 (57%) | 7.736 | 0.102 |
|  | 100000 - 200000 | 26 (28%) | 14 (29%) | 12 (26%) |  |  |
|  | 200000 - 500000 | 6 (6%) | 5 (10%) | 1 (2%) |  |  |
|  | > 500000 | 1 (1%) | 0 (0%) | 1 (2%) |  |  |
|  | Unknown | 6 (6%) | 0 (0%) | 6 (13%) |  |  |
| Age of primary caregivers | 26 - 45 | 43 (46%) | 21 (44%) | 22 (48%) | 3.308 | 0.191 |
|  | 46 - 65 | 40 (42%) | 22 (46%) | 18 (39%) |  |  |
|  | 66 - 85 | 11 (12%) | 5 (10%) | 6 (13%) |  |  |
| Cohort Origin | Original | 63 (67%) | 33 (69%) | 30 (65%) | 0.133 | 0.716 |
|  | New recruits | 31 (33%) | 15 (31%) | 16 (35%) |  |  |

Supplement Table 1 legend:

Baseline characteristics of children and young people with CP and their caregivers collected in 2021 prior to randomization. The table only includes those participating in both the baseline and follow-up assessments. Pearson chi-square test was used for comparison of group differences at statistical significance p<0.05. Monthly household income in Ugandan shillings; 1 USD = 3,500 Ugandan shillings (shs).
