# Supplementary figures and images for: Participation in activities of daily living after the Akwenda Intervention Program for children and young people with cerebral palsy in Uganda: A cluster‐randomized trial
Source: Dev Med Child Neurol. 2025 Feb 18;67(9):1195–205. doi: 10.1111/dmcn.16258 (PMC12336405; doi:10.1111/dmcn.16258)

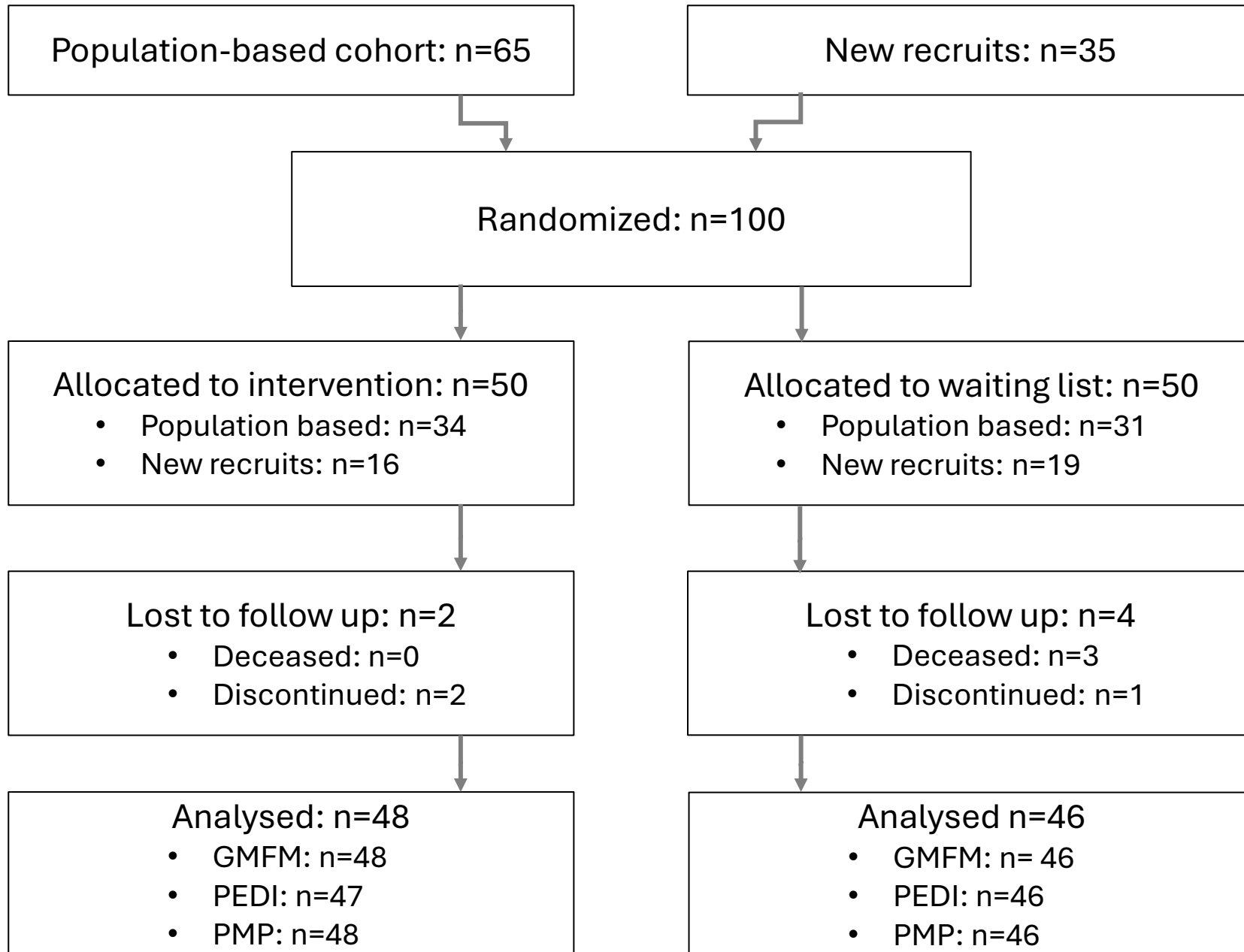

Supplement: Supplementary file 2 — Figure S1: Study flow chart. [file DMCN-67-1195-s002.pdf]
